# Retraction Note: Effectiveness of ultrasound guided dry needling in management of jumper’s knee: a randomized controlled trial

**DOI:** 10.1038/s41598-024-63509-7

**Published:** 2024-06-04

**Authors:** Faiza Sharif, Ashfaq Ahmad, Syed Amir Gilani

**Affiliations:** 1 University Institute of Physical Therapy, The University of Lahore, Lahore, Pakistan; 2 Faculty of Allied Health Sciences, The University of Lahore, Lahore, Pakistan

Retraction of: *Scientific Reports* 10.1038/s41598-023-31993-y, published online 23 March 2023

The Editors have retracted this Article.

After publication, concerns were raised about the original contribution of this work. An investigation conducted by the Editors confirmed that the Article contains material that substantially overlaps with a previous study from a partly overlapping group of authors^1^. In particular, the datasets reported in these papers are nearly identical. The Editors therefore consider this Article to be redundant.

Faiza Sharif disagrees to this retraction. Ashfaq Ahmad did not reply to correspondence from the Editors. The Editors were unable to contact Syed Amir Gilani.
